# Pyorrhea Alveolaris—What Do We Really Know of It

**Published:** 1897-05

**Authors:** W. H. Trueman


ARTICLE III.

### PYORRHEA ALVEOLARIS—WHAT DO WE REALLY KNOW OF IT.

BY DR. W. H. TRUEMAN.

This brings us to the important question, concerning pyorrhea alveolaris, What do we really know? Upon what points do those who have carefully studied it universally agree? There are, it is a satisfaction to know, a few points upon which there has been little if any controversy; and these may, perhaps, furnish us reliable data for future investigations.

1. It is a disease seldom appearing until the patient approaches middle life. While exceptions to this have been frequently noted, they are, however, infrequent and exceptional, and have been so recognized.

2. It is not, at the outset, usually accompanied by any marked systemic derangement.

3. It usually begins insidiously, runs an uneven course, and is prone to relapse.

4. It always ceases, completely and permanently, when the teeth are lost.

I note that now and again observers have remarked the close resemblance physically, and the very marked differences pathologically, between pyorrhea and that acute inflammation of the gums due entirely to systemic conditions, known as stomatitis. This is ushered in by well-marked and unmistakable systemic derangement, quickly reaches its height, responds promptly to systemic treatment, and, as a rule, in mild cases leaves the involved oral tissues but little the worse for its presence. When at its height, this condition resembles closely, so far as it affects the oral cavity, pyorrhea at its worst. We have the same turgid alveoli, the same fetid odor, and the same loosening of the teeth. Stomatitis, however, is a disease of childhood; pyorrhea, of mature age. Stomatitis comes on quickly, the acute and violent oral symptoms are merely the expression of a more profound systemic derangement —a systemic derangement so profound, indeed, as to frequently compromise life. In a mild, uncomplicated attack, it quickly runs its course, and the oral symptoms disappear as quickly as they came. It is very probable that the oral symptoms linking together stomatitis and pyorrhea are alike due to germ-infection, the sudden and violent onset in stomatitis being due to quickly-developed conditions favoring germ activity, and to the violent inflammation proving fatal to germ life. In severe cases so violent is the inflammation that gangrene, with all its attendant seriousness, not infrequently supervenes. In adults the gums are not so responsive to systemic disturbances, nor yet do slight disorders of the nutritive functions, as in early childhood, so quickly or so profoundly affect the general health. Under these conditions stomatitis becomes far less frequent and far less serious. It still, however, preserves its well marked characteristics distinguishing it from pyorrhea alveolaris, and these may have value in determining the cause and character of the latter disorder. I am strongly impressed that, after all, pyorrhea alveolaris is merely a germ-infection. It may be serious, involving a large territory, and incurable, or slight and easily controlled, just in proportion as the oral conditions favor or repress germ activity. How far the general health may affect these conditions we have, as yet, little reliable data. The pathology of stomatitis is suggestive upon this point. We may bear in mind that for the germ to become a pathological factor three things are necessary: (1) the germ; this, however, is omnipotent; (2) a dwelling-place; (3) congenial surroundings. In stomatitis the disturbed circulation, due to the primal lesion resulting in the swollen gum margins, furnishes over a widely extended territory abundant opportunity for germ colonies, and the surroundings seem particularly favorable to germ activity. Disordered digestion in the child and in the adult seems to prodnce in the oral cavity conditions especially favorable to germ-growth. In proof of this, the furred tongue, for ages recognized as a reliable and delicate indication of gastric disturbance, is now known to be due to the presence of germ life,—a form of germ-life that immediately disappears when the stomach resumes its normal condition.

In early life, while the gum-tissues firmly embrace the teeth and fully occupy the dental interspaces, there is little opportunity for any form of germ-life to obtain permanent foothold. Later, however, when normal changes materially alter this condition of affairs, when the saliva become more loaded with inorganic matter, when there is a more marked disposition to tartar deposits, when the gum tissues relax their firm embrace of the teeth and as a result debris-collecting spaces are formed along the gum-margins and between the teeth, when in addition to all this, as the natural result of normal wear and tear and the inevitable loss of tooth-substance, the gum and tooth-supporting tissues become more and more exposed to violence and accidents of various kinds during mastication, etc., we readily see how, in a thousand and one ways, opportunities for germ-infection are vastly increased. All without the slightest assistance from any systemic disorders.

We may remember, also, that of all germicides known to science none are so potent, so thoroughly reliable, or so universally accessible as are the natural secretions of a healthy human mouth. Were it otherwise, it would be utterly impossible to find a healthy individual among the many thousands who are compelled to daily breathe the germ-laden dust of a large city. It is only when the germ succeeds in quickly finding a little pouch in which to hide, secure from this, to it, destructive secretion, or when by mere chance it obtains foothold while the potency of this secretion is temporarily impaired, that it has the slightest chance to begin its destructive work. Once firmly settled, it begins to thrive, and a colony once formed creates conditions that may successfully combat its untoward surroundings. Changes constantly taking place in the oral secretions—now favoring and now opposing—fully account for the ebb and flow that marks, in its earlier stages, this disorder. The ravages of the disease, never wholly obliterated after it has once been firmly established, are ever an open door inviting the frequently noted relapses, until the complete loss of the teeth destroys forever its once favorite camping ground.

We thus see how fully the germ theory meets all of the few points upon which close observers for nearly two cedturies are in perfect accord.

I present it to your notice, not as a new theory, but as a theory fully in line with modern thought, and so fully in accord with observed facts as to warrant its selection as a new stand-point from which to study the cause and treatment of pyorrhea alveolaris,—a stand-point much to be preferred to those older theories with which, as I have shown you, our grandfathers labored so earnestly and so long, with so little profit.—*International Dental Journal.*

